# mGem: Population genomics of *Staphylococcus aureus* bacteremia and the impact of the COVID-19 pandemic

**DOI:** 10.1128/mbio.03665-25

**Published:** 2026-04-13

**Authors:** Miquel Sánchez-Osuna, Ana Beatriz Garcez Buiatte, Rebecca Wang, Oriol Gasch, Isabella W. Martin, Cheryl P. Andam, Oscar Q. Pich

**Affiliations:** 1 Laboratori de Recerca en Microbiologia i Malalties Infeccioses, Hospital Universitari Parc Taulí, Institut d’Investigació i Innovació Parc Taulí (I3PT-CERCA), Universitat Autònoma de Barcelona 16719, Sabadell, Spain; 2 Institut de Biotecnologia i Biomedicina, Universitat Autònoma de Barcelona 16719, Cerdanyola del Vallès, Spain; 3 Department of Biological Sciences, University at Albany, State University of New York 1084, Albany, New York, USA; 4 Dartmouth College Geisel School of Medicine, Lebanon, New Hampshire, USA; 5 Dartmouth-Hitchcock Medical Center 22916, Lebanon, New Hampshire, USA; 6 Servei de Malalties Infeccioses, Hospital Universitari Parc Taulí, Institut d'Investigació i Innovació Parc Taulí (I3PT-CERCA), Universitat Autònoma de Barcelona 16719, Sabadell, Spain; The Ohio State University, Columbus, Ohio, USA

**Keywords:** *Staphylococcus aureus*, bacteremia, population genomics

## Abstract

The pressure of the coronavirus disease 2019 (COVID-19) pandemic on global healthcare systems and societies was unprecedented in the modern era. Social restrictions, containment measures, and disruptions in antimicrobial prescriptions and consumption during the pandemic have been reported to alter the epidemiology of bacterial diseases, although these effects likely differed markedly between locations. Here, we compare the clinical, clonal distribution, and genomic features of *Staphylococcus aureus* bloodstream isolates before, during, and after COVID-19 in two hospitals on different continents: Parc Taulí University Hospital (Spain) and Dartmouth-Hitchcock Medical Center (USA). We hypothesize that pandemic-related environmental perturbations, such as those due to infection control practices and antimicrobial exposure, may have contributed to shifts in the diversity of circulating bacterial lineages and genomic elements. Our findings revealed changes in the distribution of low-frequency clones, antimicrobial resistance genes, and virulence factors, potentially reflecting changes in selective pressures in clinical environments.

## PERSPECTIVE

## CLINICAL EPIDEMIOLOGY OF *STAPHYLOCOCCUS AUREUS* BACTEREMIA

The burden of *Staphylococcus aureus* bacteremia (SAB) worldwide is distressingly high, with a case fatality rate ranging from 15 to 30% and an estimated 300,000 deaths per year (1). In a meta-analysis of SAB from 11 high-income countries in three continents, incidence estimates ranged from 9.3 to 65 cases/100,000/year (2). When SAB is not treated promptly (<48 h) and with the correct antimicrobial treatment, 90-day mortality risk in prolonged SAB cases reaches 39% (1). The recurrence rate is somberly high, ranging from 5 to 10% (3, 4). A major concern is that SAB causes metastatic infections, such as endocarditis and septic arthritis, in over one-third of cases, complicating treatment (1). Multidrug-resistant and methicillin-resistant *S. aureus* (MRSA) further exacerbate disease burden, worsen clinical outcomes, increase costs, and severely limit treatment options (5–7). Yet, an international standard of care for the management of SAB is lacking, and large variation in management, diagnostics, and definitions of SAB worldwide remains a major challenge (8).

The coronavirus disease 2019 (COVID-19) pandemic in 2020 imposed unprecedented operational strain on hospitals, characterized by surges in admissions, particularly to intensive care units (ICUs), and acute shortages of personnel and critical supplies. In response, institutions expanded ICU capacity, repurposed clinical spaces, and altered staffing models, resulting in higher patient-to-provider ratios and diminished infection-prevention oversight (9). These system-level disruptions were accompanied by substantial increases in nosocomial infections, especially those associated with invasive devices. Rates of central line-associated bloodstream infections (CLABSI), catheter-associated urinary tract infections (CAUTI), and ventilator-associated events (including ventilator-associated pneumonia (VAP)) rose sharply during pandemic surges, with CLABSI and VAP incidence in patients with COVID-19 reported to be severalfold higher than in non-COVID-19 cohorts (10–12).

The escalation in device-related and ICU-acquired infections was driven by prolonged hospital stays, greater reliance on invasive procedures, and erosion of standard infection-control practices due to resource constraints (13). Concurrently, hospital antibiotic consumption increased, reflecting widespread empirical use of broad-spectrum agents. In Spain, the combination of hydroxychloroquine and azithromycin began to be widely recommended for the treatment of severe COVID-19 during the first months of the pandemic, mainly between March and May 2020. This was based on preliminary studies and *in vitro* data, as well as on regional hospital protocols that included this combination for severe pneumonia (14, 15). However, this recommendation was progressively withdrawn, as results from large randomized clinical trials demonstrated a lack of benefit and an increased risk of adverse events, particularly cardiac toxicity (15–18). Most protocols reviewed up to July 2020 had already removed this combination as a recommended treatment (14). In the United States, early COVID-19 treatment trends were similar (19). After initially granting “emergency use authorization” to hydroxychloroquine in March 2020, the U.S. Food and Drug Administration revoked that authorization in June 2020 (20). Organizations such as the American College of Physicians issued clear recommendations against the use of chloroquine or hydroxychloroquine, either alone or combined with azithromycin, for the treatment of COVID-19 outside clinical trials, due to the lack of evidence of efficacy and the unfavorable safety profile (21, 22).

During the COVID-19 pandemic, SAB in patients with SARS-CoV-2 infection was marked by a substantial rise in incidence, particularly among hospitalized and critically ill individuals. Most episodes were hospital acquired and frequently linked to invasive procedures, such as mechanical ventilation and central venous catheterization. MRSA accounted for a disproportionately high share of isolates in COVID-19 patients compared with non-COVID-19 cohorts, with several studies reporting MRSA prevalence surpassing 30% in this population (10, 23). Clinical outcomes were markedly worse among patients with concurrent COVID-19 and SAB, who experienced higher rates of ICU admission, shock, respiratory failure, and death. Reported in-hospital mortality ranged from 35% to more than 60%, significantly exceeding that observed in SAB patients without COVID-19 (24–26).

## POPULATION STRUCTURE AND TEMPORAL DYNAMICS OF SAB LINEAGES

Analyses of local SAB populations suggest that COVID-19 exerted limited influence on the distribution of dominant clones, with no lineage consistently linked to SARS-CoV-2 coinfection (27). However, regional shifts in rarer clones indicate that local epidemiological pressures may have reshaped population dynamics. Notably, ST7 emerged as a predominant lineage in Wuhan following the pandemic (28), while methicillin- and mupirocin-resistant ST3390 strains were recently reported in Florida (29).

To investigate whether similar geographically driven trends occurred elsewhere, we analyzed genomic data from SAB isolates collected at Parc Taulí University Hospital (PTUH; Spain) (30) and Dartmouth-Hitchcock Medical Center (DHMC; USA) (31). To enable cross-continental comparison, we focused on isolates obtained between 2014 and 2022, comprising 339 from PTUH and 805 from DHMC. Isolates collected from March 2020 onward, corresponding to the onset of lockdown measures, were classified as pandemic clones. A full version of the computational methods used is available in Data S1. As this study is based on observational genomic data from two institutions, the results reflect population-level associations, not direct causal relationships. Cross-site comparisons must be interpreted with caution given the considerable differences in healthcare practices, pandemic burden, and antimicrobial stewardship between Spain and the United States.

**Fig 1:**
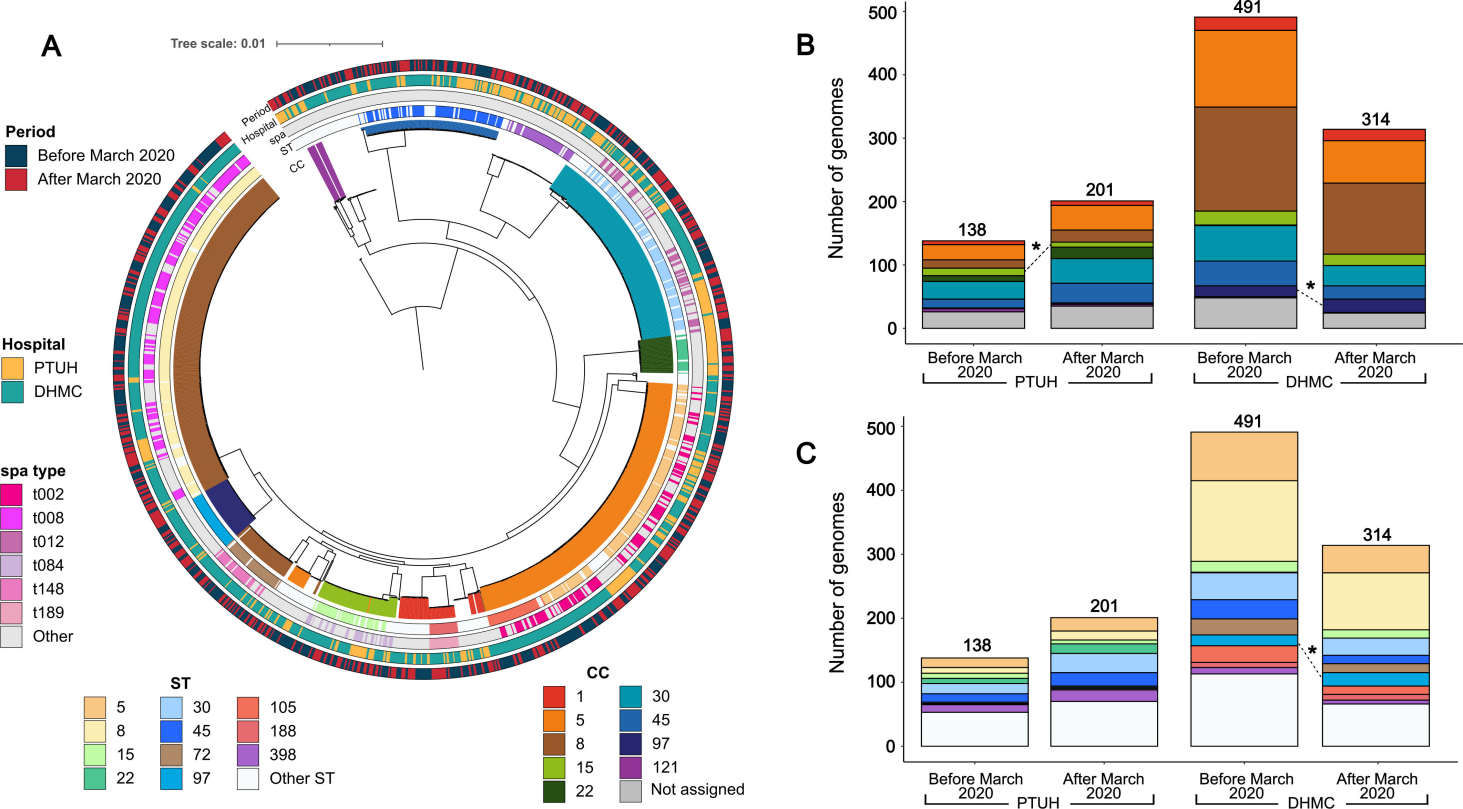
Phylogenetic relationship and population structure of 1,144 SAB genomes from PTUH and DHMC isolated between 2014 and 2022. (**A**) Maximum likelihood (ML) core genome phylogenetic tree of SAB genomes from PTUH and DHMC. Outer rings (from inside to outside) show the sequence type (ST), *spa* type, hospital, and period. Branches are colored according to clonal complex (CC). The tree is built from 166,259 core SNPs and midpoint rooted. (**B**) Distribution of CCs in each hospital before and after March 2020. (**C**) Distribution of STs in each hospital before and after March 2020. For visual clarity, only the most common STs and *spa* types are color coded, and low-abundance STs/*spa* types are grouped into “Other.” For panels** B** and **C**, the color scheme of CCs/STs is identical to that in panel A. Asterisks indicate CCs/STs that showed statistically significant differences when comparing the two periods (Fisher’s exact test, *P* < 0.05).

Phylogenetic analysis revealed that PTUH and DHMC genomes were intermingled, indicating no clear phylogeographic separation between the two populations (Fig. 1A). The most frequent clonal complexes (CCs) observed across the data set were CC8 (26.8%), CC5 (21.4%), CC30 (13.4%), and CC45 (10.2%) (Data S1). These frequencies were influenced by the overrepresentation of DHMC genomes (805/1,144; 70.4%). At PTUH, ST9968 and CC15 were significantly associated with the pre-pandemic period, whereas at DHMC, ST97 and CC97 were linked to the pandemic period (Fig. 1B and C; Table S1). Despite these associations, the overall distribution of dominant lineages remained stable, suggesting that changes primarily affected the prevalence of low-frequency clones.

These findings are consistent with previous reports of regional shifts in rarer *S. aureus* lineages (28, 29) and highlight how subtle, location-specific pressures could contribute to the emergence of new lineages and/or increased prevalence of rare lineages. Continued genomic and microbiological surveillance will be essential to monitor these dynamics and evaluate their long-term clinical and epidemiological consequences.

## GENETIC DETERMINANTS OF ANTIMICROBIAL RESISTANCE AND VIRULENCE

Different *S. aureus* strains harbor distinct combinations of genes that define antimicrobial resistance, host-pathogen interactions, and virulence potential. Our previous study identified several genetic features whose prevalence shifted during the COVID-19 pandemic (30), suggesting altered selective pressures in clinical environments. To determine whether these trends were consistent across geographic and healthcare settings, we compared the prevalence of predicted antimicrobial resistance genes (ARGs) and virulence factors (VFs) between pre-pandemic and pandemic *S. aureus* isolates from PTUH and DHMC.

**Fig 2:**
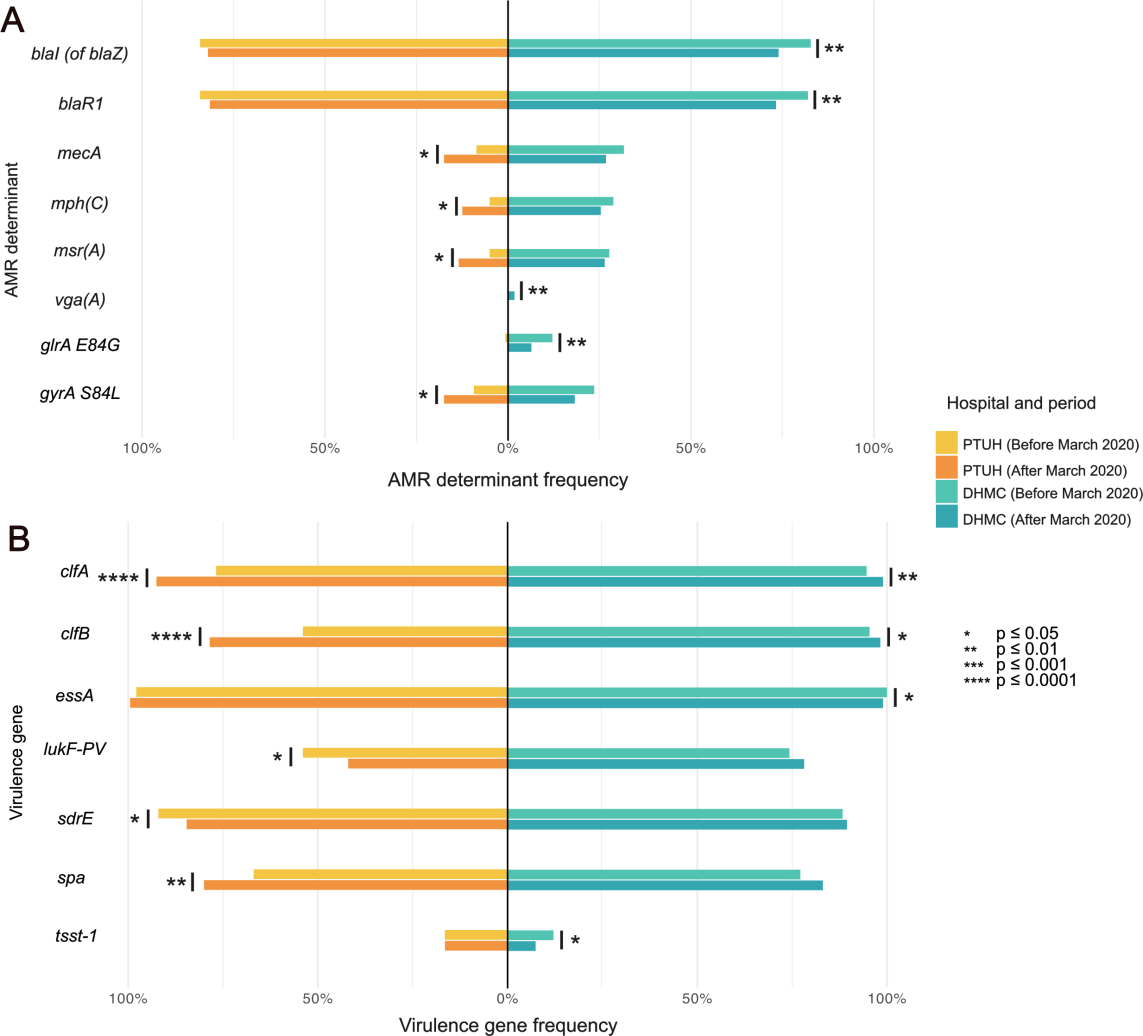
Shifts in significant (**A**) antimicrobial resistance genes and (**B**) virulence factors after March 2020 in PTUH and DHMC. This panel displays only the determinants that exhibited a statistically significant difference for PTUH, DHMC, or both institutions (Fisher’s exact test, *P* < 0.05). The full analysis is available in Table S1.

Analysis of ARGs revealed distinct resistance profiles between the two hospital populations between the pre-pandemic and pandemic periods (Fig. 2A; Table S1; Data S1). Among PTUH isolates, pandemic strains exhibited higher frequencies of the methicillin-resistance gene *mecA*, the macrolide-resistance genes *mph(C)* and *msr(A)*, and the *gyrA* S84L mutation conferring ciprofloxacin resistance. This trend is consistent with recent findings from Brazil, where increased resistance to methicillin, clindamycin, and erythromycin was observed among SAB isolates during the pandemic, with macrolide resistance associated with a higher prevalence of the *erm(C)* gene (32, 33). In contrast, DHMC isolates exhibited a significant enrichment in the lincosamide resistance gene *vga(A)*, along with a decrease of the β-lactam resistance genes *blaI* and *blaR1*, as well as the *grlA* E84G mutation associated with ciprofloxacin resistance.

These divergent patterns in the prevalence of ARGs and VFs appear to differ between the two institutions, likely reflecting local differences in the management of SAB during the pandemic period. During the first year of the pandemic, PTUH experienced major shifts in case mix, including a surge in admissions for severe COVID-19, coinciding with substantial changes in prescribing patterns. Azithromycin consumption reached an unprecedented peak in 2020 (4.74 DDD/100 hospital stays) (Table S3), more than doubling values from any other year. This sharp, isolated spike stands out clearly against overall antibiotic-use trends and is consistent with the exceptional prescribing practices of early pandemic months, which were influenced by preliminary therapeutic assumptions and rapidly evolving clinical protocols. Penicillins, cephalosporins, carbapenems, quinolones, and other antibiotic groups exhibit relatively stable year-to-year patterns. In contrast, at DHMC, no comparable spike was observed in the use of any single antimicrobial agent in 2020 compared to the pre-pandemic years (Table S4), although the institution similarly experienced a shift in case mix with increased admissions for severe COVID-19. Use of intravenous azithromycin increased 28% from 2019 to 2020, but overall azithromycin use remained stable throughout the study period. Clindamycin and ciprofloxacin use steadily declined from 2018 to 2023, while levofloxacin use remained stable. Beta-lactam use was variable across agents. However, given the observational nature of this study, we cannot directly attribute genomic changes to specific antimicrobial-use practices.

Analysis of VFs revealed consistent shifts among pandemic-period isolates, characterized by marked increases in clumping factor genes and concurrent decreases in selected toxin- and adhesion-associated genes across both hospitals (Fig. 2B; Table S1; Data S1). In PTUH isolates, the prevalence of *clfA*, *clfB*, and *spa* increased, whereas *lukF* and *sdrE* decreased. Similarly, in DHMC isolates, *clfA* and *clfB* frequencies rose during the pandemic, while *essA* and *tsst-1* declined. The *clfA* and *clfB* genes encode clumping factors that mediate fibrinogen binding and bacterial aggregation (34, 35). Within the COVID-19 context, increased fibrinogen levels and systemic immune dysregulation associated with SARS-CoV-2 infection could potentially favor *S. aureus* strains harboring these determinants (36) while diminishing the benefit of other toxin- or adhesion-related genes. Although this interpretation is speculative, it is consistent with a previous report of a shift toward SAB isolates exhibiting reduced cytotoxicity in SARS-CoV-2-infected patients (27). Collectively, these findings suggest a possible adaptation to a fibrinogen-rich, immune-modulated host environment by retaining VFs that enhance aggregation and bloodstream persistence while shedding those that confer limited advantage. The parallel VF shifts observed across both hospitals are likely driven by host-pathogen interactions rather than differences in antimicrobial stewardship or local prescribing practices.

To determine whether these genetic trends were transient or sustained, we further analyzed ARG and VF prevalence across three temporal intervals: pre-pandemic (before March 2020), pandemic (March–December 2020), and post-pandemic (from January 2021 onward). In DHMC isolates, *blaI*, *blaR1*, and *vga(A)* decreased following a transient pandemic-associated rise, whereas *clfA* frequency continued to increase in the post-pandemic period. Similarly, *clfA*, *clfB*, and *spa* were less prevalent in pre-pandemic PTUH isolates, but increased during and after the pandemic (Fig. S1; Table S2). These patterns suggest that elevated ARG prevalence during the pandemic may have been transient and potentially associated with temporary changes in antimicrobial prescribing practices (37–39). In contrast, the sustained enrichment of clumping factor genes indicates a possible adaptive fixation of VF traits favored by ongoing co-circulation of *S. aureus* and SARS-CoV-2, which continues to occur at notable incidence (40).

## CONCLUSIONS AND FUTURE DIRECTIONS

We observed significant differences in the frequency of low-frequency clonal lineages and the composition of ARGs and VFs among SAB isolates collected before, during, and after the COVID pandemic at PTUH and DHMC. These findings may be partly related to pandemic-related perturbations, such as infection control practices and antimicrobial exposure, which may have influenced the population dynamics of SAB isolates during this period. We present these findings as hypothesis-generating observations that document how a major global event may have influenced bacterial pathogen populations. However, additional data, including those from other countries and other disease types, are needed to support our hypothesis. Continued genomic and microbiological surveillance will be essential to monitor these dynamics and evaluate their long-term clinical and epidemiological consequences.

## Data Availability

The data set supporting the conclusions of this article is included within the article and its supplemental files. Genome sequence data and accession numbers of *S. aureus* are publicly available in the Short Read Archive database of the National Center for Biotechnology Information (NCBI) and published in references 30 and 31.

## SUPPLEMENTAL MATERIAL

Data S1 mbio.03665-25-s0001.txt JSON-formatted file, including the isolation information and predicted genomic data for all *S. aureus* strains included in this study.

Supplemental material mbio.03665-25-s0002.pdf Supplemental methods, Figure S1, and legends for Data S1 and Tables S1–S4.

Supplemental tables mbio.03665-25-s0003.xlsx Tables S1–S4.

ASM does not own the copyrights to Supplemental Material that may be linked to, or accessed through, an article. The authors have granted ASM a non-exclusive, world-wide license to publish the Supplemental Material files. Please contact the corresponding author directly for reuse.
