# Supplementary material for: mGem: Population genomics of Staphylococcus aureus bacteremia and the impact of the COVID-19 pandemic
Source: mBio. 2026 Apr 13;17(5):e03665-25. doi: 10.1128/mbio.03665-25 (PMC13170315; doi:10.1128/mbio.03665-25)
Supplement: Supplemental material — Supplemental methods, Figure S1, and legends for Data S1 and Tables S1–S4. [file mbio.03665-25-s0002.pdf]

mGem: Population genomics of *Staphylococcus aureus* bacteremia and the impact of the COVID-19 pandemic

Miquel Sánchez-Osuna, Ana Beatriz Garcez Buiatte, Rebecca Wang, Oriol Gasch, Isabella W. Martin, Cheryl P. Andam, Oscar Q. Pich

SUPPLEMENTARY MATERIAL

Materials and Methods

Selected genomes were typed using MLST (1) and SpaTyper (https://github.com/HCGB-IGTP/spaTyper); and antimicrobial resistance genes (ARGs) and virulence factors (VFs) were predicted with abricate (https://github.com/tseemann/abricate) against the in-built NCBI AMRFinderPlus database (2) and Virulence Factor Database (3), respectively. Core SNPs were called with Snippy (https://github.com/tseemann/snippy) against the *Staphylococcus aureus* NCTC 8325 reference genome (GCF_000013425), and a Maximum Likelihood (ML) core genome phylogenetic tree was built using IQ-TREE 2 (4) with 1,000 bootstrap replicates and the TVM+F+I+G4 substitution model deduced by ModelFinder (5).
To assess lineage dynamics during the COVID-19 pandemic, isolates collected from March 2020, corresponding to the onset of lockdown measures, were classified as pandemic clones. Fisher's exact test was applied to identify significant differences relative to pre-pandemic strains.

To determine whether these genetic trends were transient or sustained, we further analyzed ARG and VF prevalence across three temporal intervals: pre-pandemic (before March 2020), pandemic (March-December 2020), and post-pandemic (from January 2021 onward). Chi-square tests were used to assess differences across periods, followed by Bonferroni-corrected post hoc analyses.

References

1. Larsen MV, Cosentino S, Rasmussen S, Friis C, Hasman H, Marvig RL, Jelsbak L, Sicheritz-Pontén T, Ussery DW, Aarestrup FM, Lund O. 2012. Multilocus sequence typing of total-genome-sequenced bacteria. J Clin Microbiol 50:1355–1361.
2. Feldgarden M, Brover V, Haft DH, Prasad AB, Slotta DJ, Tolstoy I, Tyson GH, Zhao S, Hsu C-H, McDermott PF, Tadesse DA, Morales C, Simmons M, Tillman G, Wasilenko J, Folster JP, Klimke W. 2019. Validating the AMRFinder Tool and Resistance Gene Database by Using Antimicrobial Resistance Genotype-

Phenotype Correlations in a Collection of Isolates. Antimicrob Agents Chemother 63:e00483-19.
3. Liu B, Zheng D, Zhou S, Chen L, Yang J. 2022. VFDB 2022: a general classification scheme for bacterial virulence factors. Nucleic Acids Res 50:D912–D917.
4. Minh BQ, Schmidt HA, Chernomor O, Schrempf D, Woodhams MD, von Haeseler A, Lanfear R. 2020. IQ-TREE 2: New Models and Efficient Methods for Phylogenetic Inference in the Genomic Era. Mol Biol Evol 37:1530–1534.
5. Kalyaanamoorthy S, Minh BQ, Wong TKF, von Haeseler A, Jermiin LS. 2017. ModelFinder: fast model selection for accurate phylogenetic estimates. Nat Methods 14:587–589.

Supplementary Figures, Data, Tables

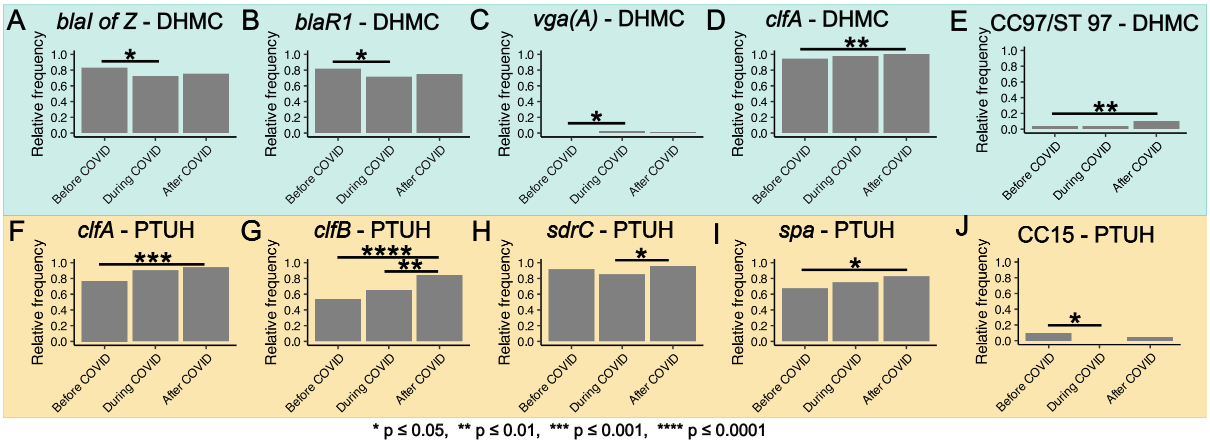

Figure S1: Temporal shifts of significant antimicrobial resistance genes (ARGs), virulence factors, and lineages across pre-, during, and post-COVID-19 time periods. Significantly altered (A-C) ARGs, (D) VFs and (E) ST/CC for DHMC. Significantly altered (F-I) virulence genes and (J) CC for PTUH. Only features with statistically significant differences (Chi-square test, $p < 0.05$) between the epidemiological periods are shown. Full statistical results are available in Table S2.

Data S1: JSON-formatted file including the isolation information and predicted genomic data for all *S. aureus* strains included in this study.

Table S1: Association tests between *S. aureus* ST/CC, *spa* type, and predicted antimicrobial resistance genes (ARGs) and virulence factors (VFs) in both PTUH and DHMC hospitals across the pre-pandemic and pandemic periods using Fisher's exact test.

Table S2: Association tests between *S. aureus* ST/CC, *spa* type, and predicted antimicrobial resistance genes (ARGs) and virulence factors (VFs) in both PTUH and

DHMC hospitals across the pre-pandemic, pandemic, and post-pandemic periods using Chi-square and post-hoc testing.

Table S3: Annual antibiotic consumption at Parc Taulí University Hospital (2008-2024), expressed as Defined Daily Doses (DDD) per 100 hospital stays.

Table S4: Antimicrobial use trends for selected antimicrobial agents at Dartmouth-Hitchcock Medical Center (New Hampshire, USA) from 2017 to 2024 expressed as antimicrobial days / 1000 days present.
